# Supplementary material for: Oxidative Stress and Extracellular Matrix Remodeling Are Signature Pathways of Extracellular Vesicles Released upon Morphine Exposure on Human Brain Microvascular Endothelial Cells
Source: Cells. 2022 Dec 4;11(23):3926. doi: 10.3390/cells11233926 (PMC9741159; doi:10.3390/cells11233926)
Supplement: Supplementary file 1 [file cells-11-03926-s001.zip › cells-1916128-SM Figures.pdf]

Supplemental Figure S1

A.

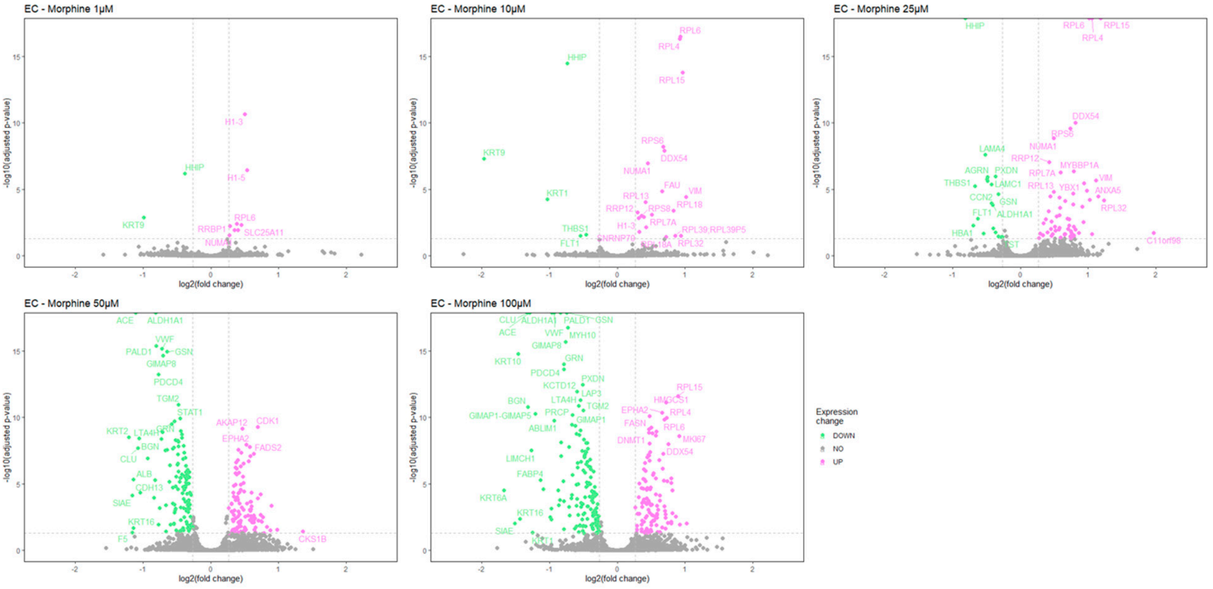

B.

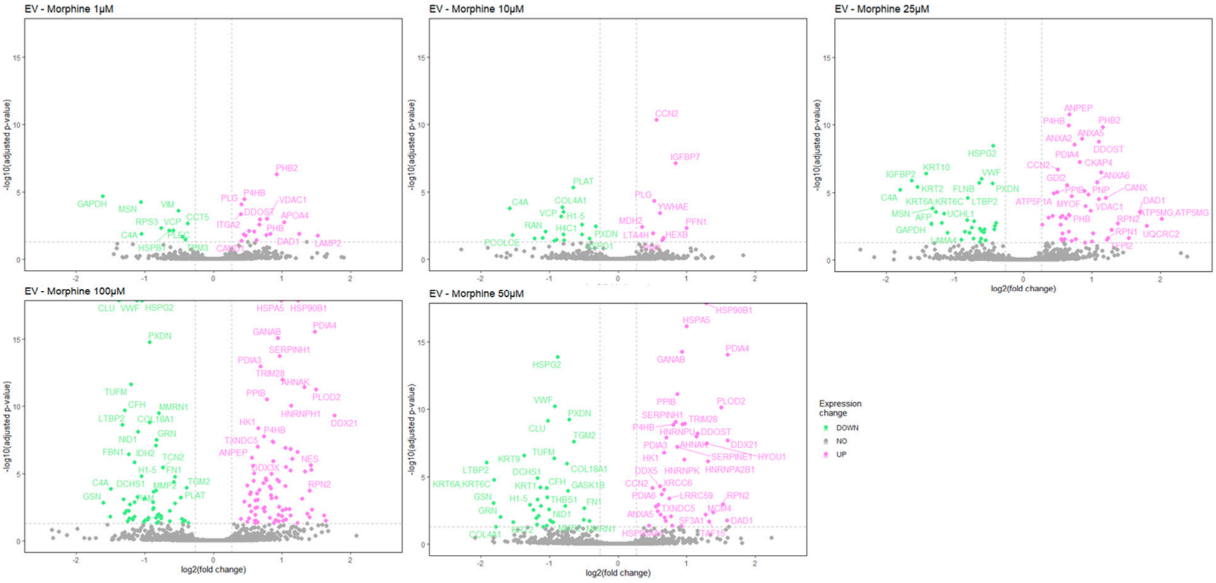

Figure S1. Volcano plots. (A) Volcano plots displaying quantified proteins from morphine-treated human brain endothelial cells at 1, 10, 25, 50 and 100µM and **(B)** from EVs released by morphine-treated human brain endothelial cells. Grey dots correspond to proteins which

are not differentially expressed. Green dots correspond to downregulated proteins. Pink dots correspond to upregulated proteins. Horizontal dot line represents cut at $-\log_{10}(p\text{-value}) = 1.33$ as p-value = 0.05. Vertical dot lines represent cut at $\log_2(\text{fold-change}) = -0.26$ and 0.26 as fold change is set at 1.2.

Supplemental Figure S2

A.

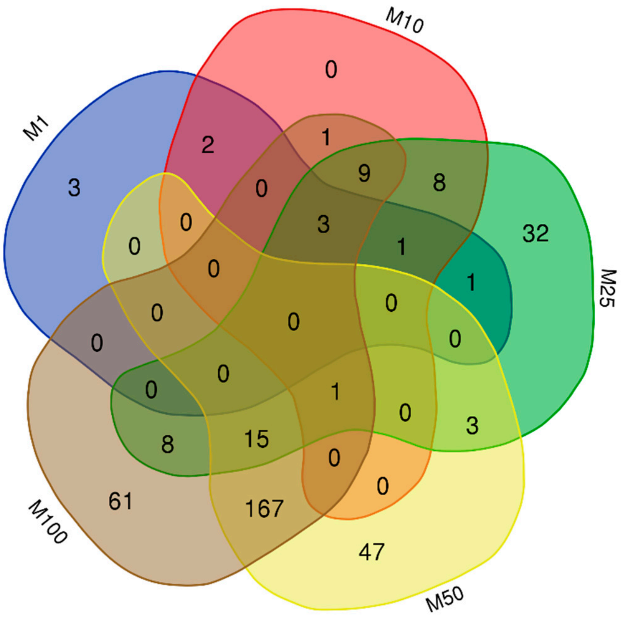

B.

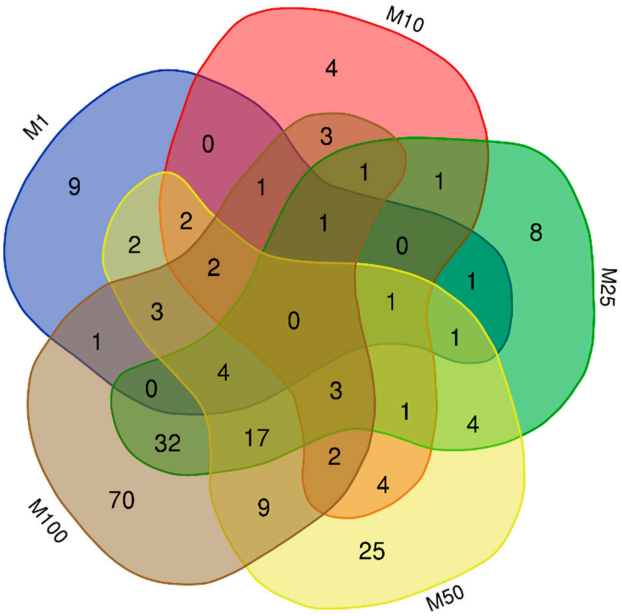

C.

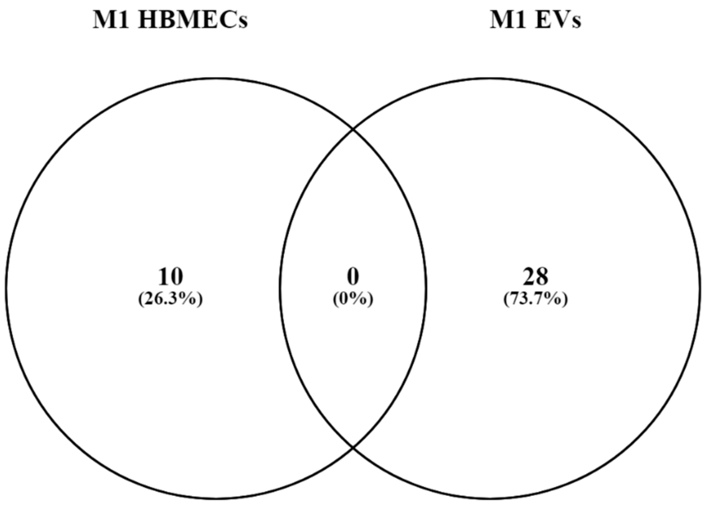

D.

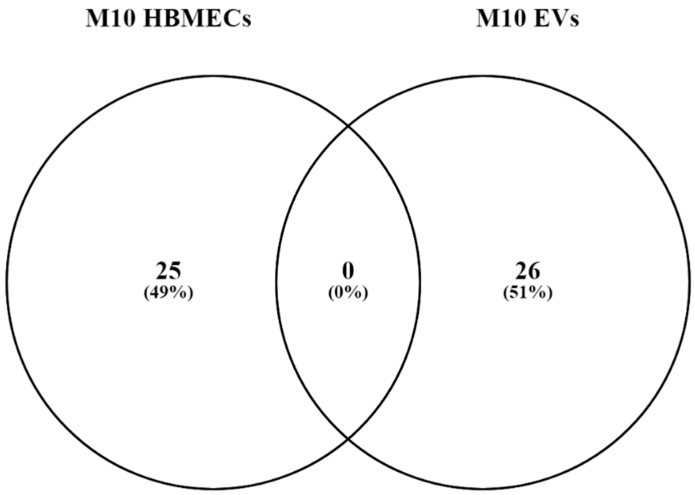
M10 HBMECs
M10 EVs
25
(49%)
0
(0%)
26
(51%)

E.

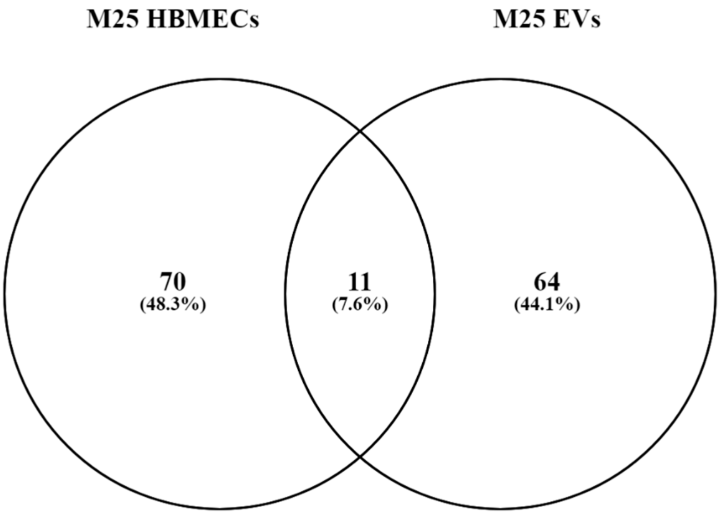
M25 HBMECs
M25 EVs
70
(48.3%)
11
(7.6%)
64
(44.1%)

F.

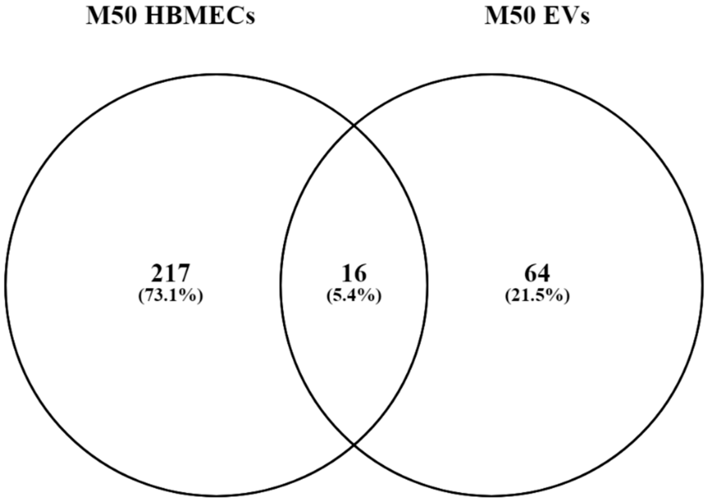
M50 HBMECs
M50 EVs
217
(73.1%)
16
(5.4%)
64
(21.5%)

G.

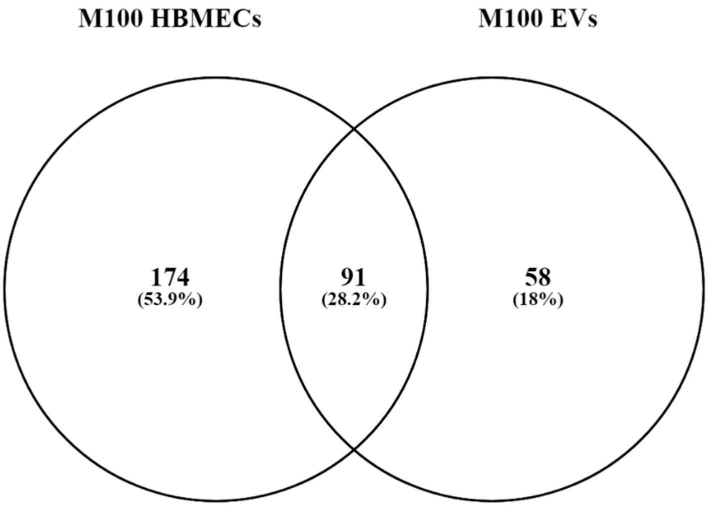

Figure S2. Venn diagrams of quantified proteins in (A) in HBMECs for morphine condition at 1, 10, 25, 50 and 100 µM, (B) derived EVs for morphine condition at 1, 10, 25, 50 and 100 µM, (C) HBMECs and derived EVs for morphine condition at 1 µM, (D) at 10 µM, (E) at 25 µM, (F) 50 µM and (G) at 100 µM. Venn diagrams were created with https://bioinfogp.cnb.csic.es/tools/venny/index.html and https://bioinformatics.psb.ugent.be/webtools/Venn/.
