# Supplementary material for: Oxidative Stress and Extracellular Matrix Remodeling Are Signature Pathways of Extracellular Vesicles Released upon Morphine Exposure on Human Brain Microvascular Endothelial Cells
Source: Cells. 2022 Dec 4;11(23):3926. doi: 10.3390/cells11233926 (PMC9741159; doi:10.3390/cells11233926)
Supplement: Supplementary file 1 [file cells-11-03926-s001.zip › cells-1916128-supplementary File S1.pdf]

Metabolomic methodology

For metabolomics analyses, HBMECs were exposed in triplicate to 0, 5, 50, and 100 µM of morphine for 24 h. Cells were scraped after quenching metabolism with 1 mL of ice-cold methanol containing 1 µM of technical internal standards CHES and HEPES (Sigma-Aldrich, St. Louis, USA) [1] and stored in 1.5 mL Eppendorf tubes at -80°C until assayed. Pooled quality controls (QCs) were prepared by mixing equal amounts of each sample. Four hundred µL of samples and QCs were evaporated to dryness for 2 hours using a SpeedVac (SC210 A, Thermo Fisher Scientific, Waltham, MA, USA), reconstituted in 80 µL of isopropanol:water (80:20 v/v), vortexed for 10 s, mixed at 700 rpm for 15 min at 4 °C (Thermomixer C, Eppendorf, Hamburg, Germany), and centrifuged at 16,230 g for 15 min at 4 °C (5810 R, Eppendorf). A volume of 70 µL was transferred to a LC vial and 5 µL was injected for analysis.

The metabolomics profiling was based on Pezzatti *et al.* [2] using a flow-through needle H-Class Acquity UHPLC system coupled to a Vion TWIMS-qTOF MS (Waters, Milford, MA, USA). Sample manager was set at 4°C and injection volume was 5 µL. HILIC separations were performed on an Atlantis Premier BEH zHILIC analytical column (2.1 x 150 mm, 1.7 µm) and with a 5 mm guard column. Solvent A was water (Optima, Fisher Scientific) containing 10 mM ammonium acetate (LiChropur, Sigma-Aldrich) adjusted to pH 9.3 with ammonium hydroxide (28 % NH3, Sigma-Aldrich) and solvent B was acetonitrile (Optima, Fisher Scientific). MS analysis was performed in negative electrospray ionization mode over a mass range of 50 – 1000 m/z with a scan time of 0.5 s. The source parameters were as follows: capillary energy: -0.6 kV; cone voltage: 40 V; source temperature: 120 °C; desolvation temperature: 500 °C; cone and desolvation gas flows were 50 and 800 L/h, respectively. Analysis was performed in MSE acquisition mode (ramp 15 – 60 V). Lock mass was leucine-enkephalin (m/z 554.2615 in negative mode) infused at 1 min intervals. UNIFI v.1.9.3 software (Waters) was used for instrument control and data acquisition.

Raw files were exported to MassLynx v. 4.1 (Waters) for file conversion to .raw format and centroiding. Then, files were further converted to .mzML format and lock mass scans removed using ProteoWizard [3]. Quality of the acquisitions was checked using MZmine 2.53 [4] and pre-processing was done in MS-DIAL 4.70 [5], following the workflow of Meister *et al*.[1]. Identifications were based on an in-house compound library containing 597 compounds [2]. A total of 104 compounds were annotated: 51 based on accurate mass and retention time (AMRT) and 53 on accurate mass (AM) only. Peak areas exported from MS-DIAL were MS drift corrected [6] and normalized by probabilistic quotient normalization using QC medians.

1. Meister, I.; Zhang, P.; Sinha, A.; Sköld, C.M.; Wheelock Å, M.; Izumi, T.; Chaleckis, R.; Wheelock, C.E. High-Precision Automated Workflow for Urinary Untargeted Metabolomic Epidemiology. *Anal Chem* **2021**, *93*, 5248-5258, doi:10.1021/acs.analchem.1c00203.
2. Pezzatti, J.; González-Ruiz, V.; Codesido, S.; Gagnebin, Y.; Joshi, A.; Guillarme, D.; Schappler, J.; Picard, D.; Boccard, J.; Rudaz, S. A scoring approach for multi-platform acquisition in metabolomics. *Journal of Chromatography A* **2019**, *1592*, 47-54, doi:https://doi.org/10.1016/j.chroma.2019.01.023.
3. Kessner, D.; Chambers, M.; Burke, R.; Agus, D.; Mallick, P. ProteoWizard: open source software for rapid proteomics tools development. *Bioinformatics* **2008**, *24*, 2534-2536, doi:10.1093/bioinformatics/btn323.
4. Pluskal, T.; Castillo, S.; Villar-Briones, A.; Oresic, M. MZmine 2: modular framework for processing, visualizing, and analyzing mass spectrometry-based molecular profile data. *BMC Bioinformatics* **2010**, *11*, 395, doi:10.1186/1471-2105-11-395.
5. Tsugawa, H.; Cajka, T.; Kind, T.; Ma, Y.; Higgins, B.; Ikeda, K.; Kanazawa, M.; VanderGheynst, J.; Fiehn, O.; Arita, M. MS-DIAL: data-independent MS/MS deconvolution for comprehensive metabolome analysis. *Nature Methods* **2015**, *12*, 523-526, doi:10.1038/nmeth.3393.
6. Klåvus, A.; Kokla, M.; Noerman, S.; Koistinen, V.M.; Tuomainen, M.; Zarei, I.; Meuronen, T.; Häkkinen, M.R.; Rummukainen, S.; Farizah Babu, A.; et al. "notame": Workflow for Non-Targeted LC-MS Metabolic Profiling. *Metabolites* **2020**, *10*, doi:10.3390/metabo10040135.
